# Acupuncture in Practice: Investigating Acupuncturists' Approach to Treating Infantile Colic

**DOI:** 10.1155/2013/456712

**Published:** 2013-11-13

**Authors:** Kajsa Landgren

**Affiliations:** Department of Health Science, Faculty of Medicine, Lund University, P.O. Box 157, 221 00 Lund, Sweden

## Abstract

Infantile colic is common, but no safe and effective conventional treatment exists. The use of acupuncture has increased despite weak evidence. This practitioner survey explores and discusses how infantile colic is regarded and treated in Traditional Chinese Medicine (TCM). The study is based on personal communication with 24 acupuncturists from nine countries. These acupuncturists specialize in pediatric acupuncture and represent different styles of acupuncture. Their experiences are discussed and related to relevant books and articles. Informants claimed good results when treating infants with colic. The TCM patterns commonly described by informants matched the textbooks to a great extent. The most common syndromes were “stagnation of food” and “Spleen Qi Xu.” Regarding treatment, some informants followed the teachers' and the textbook authors' advice on differentiated treatment according to syndrome. The points used most often were LI4, ST36, and Sifeng. Other informants treated all infants alike in one single point, LI4. The results demonstrate the diversity of TCM. The use of acupuncture for infantile colic presents an interesting option, but further research is needed in order to optimize the effects and protect infants from unnecessary or less effective treatment.

## 1. Introduction

Infantile colic, where “an otherwise healthy infant cries and/or fusses for more than 3 hours per day, more than 3 days per week,” affects 10–40% of infants. Typically, it starts when the baby is two weeks old and often disappears spontaneously once the infant reaches 3-4 months [1]. Currently, no safe and effective medications exist and the use of acupuncture has been increasing despite weak evidence. Even if the prognosis is good, the baby and the family suffer for the duration [2, 3]. Infants with colic also have an increased risk of exposure to physical abuse [4]. For this reason, it is essential to find a safe and effective treatment that curtails the colic period.

Acupuncture can be characterized by either an ancient Asian medical tradition including a broad diversity of styles and approaches (referred to here as Traditional Chinese Medicine (TCM)) or by a modern Western medical view, where acupuncture can be described as “sensory stimulation of the nervous system.” TCM, encompassing not only acupuncture but also herbs, moxibustion, cupping, Tui Na, Tai Ji, Qi Gong, and dietary and lifestyle advice, has been practiced for thousands of years. According to Western medicine, the effect of acupuncture is explained as the release of neurotransmitters and hormones in the central nervous system [5]. Acupuncture has been used mainly to treat somatic pain. It also affects visceral pain, the autonomic system, the gastrointestinal system, anxiety, and sleep [5], indicating a possibility that acupuncture may relieve infantile colic.

An early pilot study involving 13 infants with night crying reported a positive effect from acupuncture in the points Sifeng and PC7 [6]. Similarly, another uncontrolled study using PC9 on 100 infants [7] also reported a positive effect. In a qualitative study, 67 out of 68 parents reported that colicky symptoms ceased after a mean of four acupuncture treatments [8]. Minimal acupuncture in one single point (LI4) reduced the duration and intensity of crying in two randomized trials including 40 and 81 infants, respectively [9, 10]. This use of LI4 was also associated with minor changes in frequency of bowel movement and improved sleep [11]. An ongoing study is evaluating the effects of ST 36 [12]. In a case study including 913 infants with colic who received a mean of six acupuncture treatments in LI4, parents rated colicky crying and stooling as less frequent and their infants as having an inflated Stomach less often, with a higher frequency of drooling, belching, and regurgitation [13]. There were no reports of serious side effects from acupuncture. In clinical TCM practice, acupuncture might not be given in a single point and not in the same point for all patients with a specific condition such as colic. The evaluated treatment, then, is not the treatment used in clinical practice. The aim of this practitioner survey is to investigate acupuncture as it is typically performed in practice by TCM acupuncturists. Based on personal communication with internationally recognized pediatric acupuncture teachers and with experienced practitioners who also specialize in pediatrics, this paper explores and discusses how acupuncturists trained in TCM regard and treat infantile colic.

## 2. Method

At a major acupuncture conference in Europe in 2012 at which one of the themes was pediatric acupuncture, nearly all of the speakers who lectured on pediatrics as well as acupuncturists with a wide experience of pediatric acupuncture were asked by the author to share their experience of treating infants with colic. These acupuncturists pointed out other colleagues who also treat infants with colic. They were all given an information sheet describing the aim of the interview together with a set of questions. Informants were told that guidelines for a planned trial (ClinicalTrials.gov NCT01761331) were being drawn up and that their expert opinion on the most effective way of treating infantile colic with acupuncture was being requested. Informants were asked about methods of diagnosis of infants with colic, points of frequent use, whether they preferred particular points for special symptoms such as vomiting or frequency or absence of stooling, points they considered to be inappropriate, stimulation of needles, treatment frequency, advice given to parents, and whether they used acupuncture-related therapies.

Fourteen people of those asked took the time to answer the questions and, due to lack of time, two abstained. Some interviews were short, conducted in a busy environment or directly after a long day of lecturing. Others resulted in long, lively discussions. Not all informants answered all of the questions. Notes were made during these informal discussions. Alternatively, the informants wrote their answers. After the conference, a questionnaire was emailed to a further ten acupuncturists who treat infants, asking whether they would share their clinical experience. Seven did so via email. Later, a similar survey was sent to six acupuncturists with extensive knowledge of neurophysiology. Two of these answered.

## 3. Results and Discussion

In total, 23 professionals from five European and four non-European countries shared their experience. The median experience of practicing acupuncture was 25 years. Many had performed pediatric acupuncture for more than 20 years and had extensive experience of treating infants with colic. Among the 15 informants who supplied approximate numbers, seven had treated 30–50 infants, four had treated 100–250 infants, and four had treated 900–3,000 infants each. 14 informants taught acupuncture to varying extents, and are referred to in this study as “teachers.” The two informants specializing in neurophysiology are referred to as “Western acupuncturists” and the other informants as “clinicians.” Seven had published textbooks on acupuncture and seven either held a Ph.D. or were doctoral students. There were five medical doctors, nine nurses/midwives, and one physiotherapist. All informants with the exception of one western acupuncturist had training in TCM and all worked clinically with pediatric acupuncture with the exception of the Western acupuncturists.

The informants represented different styles of acupuncture, described by informants as TCM, Japanese acupuncture, *five elements,* and/or *stems and branches,* and were found to diagnose and treat colic in different ways. They were all generally very positive regarding the outcome of TCM treatment of colic. Their experience is discussed below and related to the literature.

## 4. Diagnosis

To cure a disease, acupuncturists maintain that they must determine its root by carefully collecting data about symptoms and signs [14]. Diagnosis in adults includes tongue diagnosis, but this cannot be used for infants [14–16]. Another diagnostic tool is examination by palpation. Palpating the abdomen can supply important information about excess or deficiency [14]. One informant who is trained in Japanese acupuncture used abdominal diagnosis to differentiate between patterns. No other informants mentioned examination of infants by palpation. Four acupuncturists based their diagnoses on pulse diagnosis, while most of the interviewed clinicians and teachers claimed that it is impossible to take the pulse of a 2–12-week-old infant. The latter opinion is supported by textbooks [14–16]. Another option in diagnosing infants is examination of the vein of the index finger. While this method provides information about pathogenic factors [14, 17], it is unreliable [15]. One informant used this method. Another mentioned that a blue vein on the bridge of the nose can signify “shock during pregnancy.” The latter idea is supported by textbooks [15, 17]. According to Flaws [16], a blue vein suggests a weak Spleen, while Loo [17] maintains that a greenish vein indicates a weak Spleen.

According to TCM, both excess and deficiency can cause colicky symptoms [17, 18]. Questions about bowel movement, appetite, smell, intensity of crying, and so forth, provide useful information about the infant's internal condition, guiding the acupuncturist's choice of points [16]. Few informants claimed to have requested this information. Informants trained in acupuncture according to the *five elements *and the philosophy of *stems and branches* stated that they took into consideration the constitutional energies by birth, used pulse diagnosis, observed infants' complexions, and listened to infants' cries in order to determine each infant's constitution. These informants indicated that they never treat a symptom but instead treat holistically, with the inclusion of spiritual aspects.

In pediatric medicine, the generally accepted definition of colic is “fussing and crying more than three hours per day, more than three days per week” [1]. Neither the informants nor TCM textbooks used this definition or even a similar one [14, 15, 17, 19]. None of the informants suggested that parents keep a diary to record how much the baby cried in order to assess the duration and intensity of symptoms. Instead, they based their treatment on parents' subjective description of symptoms. This can be misleading as parents' fatigue and general state of mind tend to affect their perception of how much their infants cry. In an earlier study, less than half of the infants of parents who sought help for colic cried in fact more than normal [10]. Keeping a diary reduces the risk of unnecessary treatment and facilitates the evaluation of the treatment's effect.

## 5. Suggestions of Syndromes

From a TCM perspective, differentiation of syndromes is essential for the choice of the correct treatment [20]. The symptoms in infantile colic can correspond to several TCM patterns or syndromes. Most teachers and two clinicians based their point selection on syndrome or constitution. Informants trained in Japanese acupuncture, *five elements*, or *stems and branches* claimed that “there are no standard points and that one must take each baby's constitution into account.” After deciding which element to treat, they chose source points, mother points, and/or a command point on the meridian that they deemed correct. Conversely, five out of seven clinicians did not differentiate treatment according to symptoms but treated all infants with colic alike. Two informants with extensive clinical experience, that is, each having treated more than 1000 infants with colic, did not distinguish between syndromes and instead always used LI4.

Among those informants who differentiated patterns, the most frequently mentioned syndromes were “stagnation of food” and “Spleen Qi Xu,” which are patterns commonly described in textbooks.

### 5.1. Stagnation of Food

“Stagnation of food”—also called “accumulation of food” or “blockage of food”—is common and causes intense, loud crying, often with a sudden onset [15]. “Stagnation of food” implies that the infant has either eaten more food than the Spleen can “transport and transform,” or that the Spleen is too weak to transform even a moderate intake of food. This shi syndrome can indicate heat [14, 16, 17, 19], even if one informant had a different opinion, stating that heat cannot be present in colicky infants. If heat is present, the baby has a voracious appetite, stooling and vomiting are explosive and foul-smelling, and the abdomen is swollen and tense [14]. Only one teacher and one other informant listed “overfeeding” as a cause of colic, recommending regulation of intervals between meals. This is surprisingly few, as textbooks highlight the importance of minimizing comfort feeding [14–17, 19], and the provision of advice about lifestyle is considered important in TCM [21]. Undoubtedly, there are many advantages of breastfeeding, but infants with “stagnation of food” are often fed rather frequently [16]. According to the literature, the time between feeding sessions should be extended to at least 2-3 hours [14, 16]. Dietary advice to breastfeeding mothers is also recommended [16, 17]. Five of the 14 teachers and all but one of the clinicians stated that they recommend dietary changes for the baby or for the breastfeeding mother. Elimination of cow's milk protein was the most common advice, followed by reduction of food that produces gas (such as cabbage and peas). None of the informants asked whether the baby showed normal weight gain. This question is relevant, as infant crying can be due to hunger.

### 5.2. Spleen Qi Xu

Informants saw “Spleen Qi Xu” as common in small children. According to TCM, children differ from adults in that they have weaker organs and a weaker “Middle Jiao,” rendering them more susceptible to disease. The Spleen is considered to be responsible for the “transformation and transportation of food.” Accordingly, many gastrointestinal problems are related to disturbances in these organs and are treated by points on the Stomach and on the Spleen channel. As infants must eat a lot to gain weight and develop properly, their digestive system works at almost maximum capacity and so is easily disrupted [16, 21]. Irregular feeding, demand feeding, and overfeeding can cause an overload [14–16]. “Spleen Qi Xu” can coexist with “Ki qi xu” [17] and precede “stagnation of food.” Infants with “Spleen Qi Xu” can cry for as many hours as those with “stagnation of food” but with less intensity. They tend to be more limp and pale and have less appetite. Furthermore, it is easier to comfort infants with “Spleen Qi Xu” than those with “stagnation of food”. 

### 5.3. Cold

 “Cold” is another syndrome that can be present in colic. Compared to “Spleen Qi Xu,” the pain is more intense and its onset can be more sudden. The infant is paler, with cold hands and feet, and warmth can relieve the pain. Vomiting and stooling have a mild odour [14, 15, 19]. The informants did not mention “cold” frequently. It was considered to stem from physical cold or chemical cold brought on by medications such as paracetamol or antibiotics. Besides LI4 and ST36, acupuncturists suggested Sifeng and sometimes moxa if symptoms were intense. 

### 5.4. Physical and Psychological Trauma

Another cause of colicky symptoms suggested by informants was physical trauma. Vacuum extraction or cranial compression due to strong contractions during birth and resulting in the stagnation of qi and blood could cause headaches. In these cases, LI4 was recommended. Some informants suggested GB20 and LR3, or a referral to a chiropractor, osteopath, or craniosacral therapist, in line with Bohlayer's recommendations [19]. However, this hypothesis is contradicted by the fact that scientific studies have not found more colic among babies delivered by vacuum extraction or caesarean section [22]. Referral to a chiropractor should be questioned, as no evidence exists for the effect of spinal manipulation in infantile colic [23].

Psychological trauma before, during, or after birth, or a dysfunctional relationship with the mother [18], was not mentioned often by informants. However, according to TCM, they can cause patterns like “shock or anxiety” [19] or “fear and fright” [16]. The Heart and the Shen are thought to be involved [19]. One informant mentioned “shock during pregnancy” as one less common syndrome and used HT7 to treat Shen in such cases. Some informants pointed out the importance of discussing the events during the third month of pregnancy with the parents, as they were convinced that psychological trauma, negative thinking, or quarreling at that time could influence the baby's health after birth. In such a case, the acupuncturist suggested that the parent ask the infant for forgiveness, claiming that the colic often disappeared if they did. Only if the colic persisted after this intervention was acupuncture suggested. As parents often feel a shamed and guilty when they cannot comfort their baby, they need support during the stressful colicky period [2]. Identifying parents as the root cause of colic creates extra pressure and should be considered with great care.

### 5.5. Other Syndromes

One teacher with considerable experience in pediatrics divided colic into three syndromes: “pain with diarrhea,” “pain with constipation,” and “pain with cold.” One clinician claimed that “phlegm” was the most common pattern and, consequently, used LU7 and LU9. One textbook refers to patterns called “hot colic” and “cold colic” [16], others to “excess colic” and “deficient colic” [17, 18]. Both patterns fit with the syndromes “food stagnation with heat” and “Spleen Qi Xu with cold.” 

## 6. Points Used

There is debate as to whether the effect of acupuncture depends on the points used [24]. TCM acupuncturists claim that point specificity and the choice of points according to patients' symptoms and signs are vital. These acupuncturists are trained to choose points at every treatment session to match each patient's actual qi, yin, and yang status. According to TCM, children are more yang than adults, which indicates the rapid changeability of their state. Additionally, children's meridian system is not fully developed. This indicates that the choice of points may differ from that for adults [16, 21]. 

The points that the teachers and clinicians used most frequently on infants with “stagnation of food” or “Spleen Qi Xu” are shown in Table 1. Twenty-three different points were claimed to have effect. This could indicate that different points have different effects and that informants are adept at choosing optimal points for each baby on each specific treatment day. It could also simply indicate that acupuncture is non-specific or that less effective treatment is given in some cases.

Even if many points were mentioned, LI4 and ST36 were the most consistently used points in both “stagnation of food” and “Spleen Qi Xu.” In a major point book [25], LI4 is claimed to eliminate heat from yangming and to promote the motion of qi in the channel, but no other references are given for LI4's effect on digestive symptoms. Neither Scott [15] nor Rossi [14] list LI4 as a main point for abdominal pain or for gastrointestinal symptoms. Yet, the two informants who treated the greatest number of infants with colic always used LI4 only. This might convey that no treatment except standardized acupuncture with very light stimulation in LI4 has been scientifically tested or thus has a proven effect [9, 10]. Hypothetically, acupuncture in LI4 may increase sympathetic activity and thereby inhibit parasympathetic nerve activity. This can result in inhibition of the intestinal peristaltic movements, leading to the alleviation of the symptoms of infantile colic [9]. 

ST36 is indicated for gastrointestinal disorders and abdominal pain [14, 15]. It is described as supplementing middle jiao, regulating intestinal qi [14], and for treating diarrhea, constipation, and flatulence [25]. In studies on gastrointestinal motility, ST36 and PC6 are the major acupuncture points [26]. Informants considered ST36 to have effect in both excessively frequent and excessively infrequent stooling, which might reflect that acupuncture has a balancing effect on the function in the autonomic system [9]. 

The common use of Sifeng in “stagnation of food” (Table 1) follows recommendations from textbooks. Sifeng, an extraordinary point on the center of each of the proximal interphalangeal joint creases of the four fingers, is recommended in childhood digestive disorders [14–16]. Sifeng is believed to fortify the Spleen [19, 25, 27], free food accumulation [14, 16], and clear heat [28]. It also has a marked dispersing effect [15]. Piercing the point with a three-edged needle and squeezing for a drop of blood or clear yellow fluid is recommended [14, 25, 28]. A milder needling of Sifeng was used by the informants and may be sufficient [15, 16]. 

Points on the back (BL20, BL21), the abdomen (ST25, CV6, CV12), and the Spleen channel (SP3, SP4) are suggested in order to supplement and regulate middle jiao qi and to disperse “stagnation of food” in infants [14]. Five informants used points on the Spleen channel even although SP6 was used most frequently. Beside Sifeng, Flaws recommends using back shu points solely [16]. None of the informants used points on the back and only two used abdominal points. ST25 is the front Mu point of the large intestine that is supposed to regulate the intestines and relieve abdominal symptoms [25]. Informants who, in addition to their acupuncture training, were also trained in Western medicine, indicated the importance of choosing points in relevant segments to obtain neurological connections between the points and the digestive organs. They stressed that the purpose of the treatment was to stimulate the sympathetic system in order to reduce intestinal motility. According to this neurological reasoning, ST25, CV12, and other points in segments relevant for intestinal functions could be useful in treating colic. However, ST25 and CV12 were used by one informant only.

## 7. Points according to Certain Symptoms

Informants were asked whether they considered using certain points for the different symptoms that often accompany infantile colic. Suggested points for frequent vomiting, frequent stooling, infrequent stooling, and excessive flatulence are shown in Table 2. ST36 and LI4 were mentioned most frequently, followed by ST25, PC6, and Sifeng.

## 8. Beliefs about Certain Points

Certain points were favored by some acupuncturists but avoided by others, depending on whether the acupuncturist considered it to be tolerated well or very painful. While nine informants regularly used Sifeng, considering it to be the most effective point, one never used it due to pain and lack of evidence. Likewise, most informants recommended LI4, while one never used LI4 for fear of draining qi. Some avoided ST36 because they considered it more painful than LI4. Conversely, some preferred ST36 because they considered it less painful than LI4. One informant used ST25 as a standard point, as “it gives good results and infants do not cry when needled there.” Other informants avoided points on the Stomach for practical and safety reasons and because they considered them painful. Informants' descriptions of effect and pain response from different points thus vary considerably. This indicates the need for further evaluation of effects and pain response. 

## 9. Safety

While acupuncture seems to be a safe treatment for infants [29, 30], some of the points suggested by informants may be less appropriate for safety reasons. For example, needling in CV17 is inappropriate due to its location on the thorax, and SJ16's location on the neck could be risky for a wriggling baby. Ear acupuncture has an effect on gastrointestinal activity [31] and may work well in pediatrics, but the baby's head must be restrained, which might be traumatic. Consequently, infants' experience of ear acupuncture merits further investigation.

## 10. Few Needles and Mild Stimulation

Besides the choice of point, needle technique is considered to be important in TCM [15, 20]. An acupuncturist can needle deeply or superficially and with mild or strong manual stimulation that can be “reinforcing” or “sedating.” The needle is often stimulated to “de qi,” a needle sensation that can be described as “heaviness” or “numbness” [20]. Informants reached a consensus about using fewer needles and mild, short needle stimulation compared to methods in adult treatments. This follows recommendations [16, 21, 32]. Children were thought to respond more quickly to treatment. Most acupuncturists used 1–4 points per treatment with a range of 1–8 insertions. Retention time varied between “just in and out” and some minutes. Most informants let the needles stay in for 2–15 seconds. One clinician explained their method of leaving the needles in “until I see that the infant feels “de qi” but before it starts crying.” In general, needles were stimulated minimally (1–5 stimulations) or not at all. Two teachers stimulated the needles until they felt “de qi” in their own fingers. Another wrote that they preferred to keep the needles in “until seeing signs of sympathetic activity, such as paleness around the mouth or a slightly increased heart rate.” A few teachers and one clinician pointed out that they stimulate the needle differently depending on excess or deficiency syndromes. The informants did not use electroacupuncture.

Most teachers recommended treatment 1-2 times/week. Clinicians treated at least twice a week, and two treated daily. Infants were treated 1–8 times. Unilateral, bilateral, and diagonal treatments were equally common. One teacher never used the same point twice. 

Needle diameter varied between 0.12 and 0.25 with a median of 0.20. Needle length was usually 13 mm but varied between 0.10 mm (Pyonex needles) and 0.30 mm.

## 11. Acupuncture-Related Techniques

Six of the 14 teachers and one of the seven clinicians rarely used needles on small children. Following recommendations found in some textbooks, they preferred the noninvasive massage techniques Shonishin, Tuina, and Guasha for treatment during early infancy [14, 16, 21]. Shonishin is related to Japanese acupuncture and replaces needles with a blunt tool used to “tap” or “stroke” the acupuncture point [21]. Informants considered these treatments to be very light and simple yet effective. Eight recommended ordinary baby massage, while one stated that massage has no effect on visceral pain. Two pointed out that teaching parents infant massage can strengthen the bond between them and their babies. One clinician used laser and magnetic beads on ear Shen Men for 20 minutes. One informant often recommends herbal medicine and fennel seed tea, as per Flaws' recommendations [16].

According to TCM, infants diagnosed with “cold” benefit from moxa treatment, which in this case means burning a moxa cigar above points located on the belly [14–16, 19]. Some informants used moxa, often on CV12, if cold was present. However, one teacher strongly discouraged using moxa on infants “due to their delicate skin” and recommended the use of a hot water bottle instead. The risks of using moxa on infants and the effect of hot water bottles or bags with qi-moving herbs on the baby's belly, used by one informant, require further investigation.

## 12. Strengths and Limitations

The author of this study (KL) has obtained an insight into how infantile colic is regarded in TCM by reading available textbooks on pediatric acupuncture and by attending courses in pediatric acupuncture (eight days with Julian Scott in Oslo in 2001, a distance learning course with Julian Scott and Teresa Barlow in 2001, four days with Alex Tiberi in Rothenburg in 2001). One strength of the present study is that KL has extensive experience in treating infants with colic and conducts research on this topic. Practitioner survey is a recommended method to gain understanding [33]. In this practitioner survey, acupuncturists from nine countries representing many traditions shared their experiences and clinical expertise, which was mirrored against textbooks to deepen the understanding of how colic is treated in TCM. Limitations are that misunderstandings might have arisen when the informant and the interviewer had different native languages and/or proficiency levels in their common language, and that all informants did not answer all questions. As a result, not all experiences are discussed.

## 13. Conclusions

Infantile colic is common, and no safe and effective conventional treatment exists. Consequently, the use of acupuncture is an interesting option. This paper shows, for the first time, that acupuncture is used for colic in at least nine countries and describes current clinical practices including point selection and details of needling. 

The results of this study exemplify the diversity of methods in TCM. Informants represent many styles of acupuncture and all claim good results when treating infants with colic. The extent to which the treatment was individualized varied, ranging from none at all (all patients received the same treatment in LI4 at all sessions) to fully individualized treatment protocols that focused on specific needs and symptoms. The TCM patterns commonly described by informants matched the textbooks to a great extent, unlike in [34, 35]. It would be interesting to investigate interrater reliability of the TCM patterns, as they have been low for another diagnosis [36]. 

Acupuncture is used in colic despite weak evidence [37], indicating that further research about effect and safety is needed. To protect infants from unnecessary or less effective treatment, research on the effect of specific points, on combination of points, on ear acupuncture, and on the difference between unilateral contra bilateral needling is needed. For maximal effect, the optimal interval between treatments should be determined. Clinically used, noninvasive, and acupuncture-related treatments, such as ear seeds, Shonishin, and Tuina, should be investigated. If non-invasive methods have the same effect, they should be recommended. 

Crying and accompanying symptoms should be recorded in clinical practice, such as in a baby diary, so that the acupuncturists know what they are treating and whether the intervention has effect. This description of how experienced practitioners use acupuncture for infantile colic is a valuable supplement to existing knowledge and can encourage reflection on everyday clinical practice.

## Figures and Tables

**Table 1:** Suggestion of points by teachers and clinicians for the two most common syndromes in infants with colic. Some informants did not suggest any points.

Points suggested	Stagnation of food		Spleen Qi Xu	
	Teachers (*n* 14)	Clinicians (*n* 7)	Teachers (*n* 14)	Clinicians (*n* 7)
Sifeng	4	5		1
LI4	5	7	2	7
ST36	2	5	3	4
LR3	1	2		2
ST40	1	1		1
ST25		1		1
HT7		1		1
SP6		2	1	1
CV12	1			
LI10	1			
LR8	2			
HT3	1		1	1
SP15			1	
KI10	1			
Yintang	1			1
SJ6	1			
SJ16		1		
Ear Shen Men				1
SP9				1
SP3 + LU9				1
LI11 + ST44		1		

**Table 2:** Points suggested by the 23 informants for frequent vomiting, frequent stooling (defined as more than 8 times per day), infrequent stooling (defined as “more than 4 days between”), and excessive flatulence in infants with colic.

	Frequent vomiting	Frequent stooling	Infrequent stooling	Excessive flatulence
Points				
Sifeng		1	2	
LI4	1	2	3	
ST36	3	6	4	1
ST25		1	3	1
ST37		1		
ST40			1	
ST45			1	
SP3		1	1	
SP15			1	
PC6	3			1
LR3		1		1
LR8				1
CV6 (moxa)				1
CV12	1			1
CV17	1			1
PC7		1		
BL25		1	1	
SJ5			1	
SJ6		1	1	
SJ16		1		
GB37/39				1
KI10				1
LU7			1	
Ear Shen Men	1			
∗		1		
No special point	5	5	3	12

*“A point on the neck, I do not remember the name. It is a forbidden point.”

## References

[1] Landgren K, Hallström I, Lindberg T (2009). Tremånaderskolik. *Pediatrisk Omvårdnad*.

[2] Landgren K, Hallström I (2011). Parents’ experience of living with a baby with infantile colic—a phenomenological hermeneutic study. *Scandinavian Journal of Caring Sciences*.

[3] Landgren K, Lundqvist A, Hallström I (2012). Remembering the chaos—but life went on and the wound healed. A four year follow up with parents having had a baby with infantile colic. *The Open Nursing Journal*.

[4] Talvik I, Alexander RC, Talvik T (2008). Shaken baby syndrome and a baby’s cry. *Acta Paediatrica*.

[5] Carlsson C (2002). Acupuncture mechanisms for clinically relevant long-term effects—reconsideration and a hypothesis. *Acupuncture in Medicine*.

[6] Liu HR (1994). Night crying in infants treated by acupuncture. *Journal of Chinese Medicine*.

[7] Zhao J (2002). Treatment of infantile morbid night crying by acupuncture at Zhongchong point in 100 cases. *Journal of Traditional Chinese Medicine*.

[8] Landgren K, Hallström I (2005). Akupunkturbehandling vid spädbarnskolik—föräldrars upplevelser av barnets beteende före och efter behandling. *Vård i Norden*.

[9] Reinthal M, Andersson S, Gustafsson M (2008). Effects of minimal acupuncture in children with infantile colic—a prospective, quasi-randomised single blind controlled trial. *Acupuncture in Medicine*.

[10] Landgren K, Kvorning N, Hallström I (2010). Acupuncture reduces crying in infants with infantile colic: a randomised, controlled, blind clinical study. *Acupuncture in Medicine*.

[11] Landgren K, Kvorning N, Hallström I (2011). Feeding, stooling and sleeping patterns in infants with colic—a randomized controlled trial of minimal acupuncture. *BMC Complementary and Alternative Medicine*.

[12] Skjeie H, Skonnord T, Fetveit A, Brekke M (2011). A pilot study of ST36 acupuncture for infantile colic. *Acupuncture in Medicine*.

[13] Reinthal M, Lund I, Ullman D, Lundeberg T (2011). Gastrointestinal symptoms of infantile colic and their change after light needling of acupuncture: a case series study of 913 infants. *Chinese Medicine*.

[14] Rossi E (2011). *Pediatrics in Chinese Medicine*.

[15] Scott J (1986). *Acupuncture in the Treatment of Children*.

[16] Flaws B (2006). *A Handbook of TCM Pediatricsed*.

[17] Loo M (2002). *Pediatric Acupuncture*.

[18] Loo M (2009). *Integrative Medicine for Children*.

[19] Bohlayer R (2008). *Chinesische Medizin für Kinder und Jugendliche: Mit psychosomatischen Aspekten*.

[20] Birch S, Felt R (1999). *Understanding Acupuncture*.

[21] Birch S (2011). *Shonishin: Japanese Pediatric Acupuncture*.

[22] Talachian E, Bidari A, Rezaie MH (2008). Incidence and risk factors for infantile colic in Iranian infants. *World Journal of Gastroenterology*.

[23] Gleberzon BJ, Arts J, Mei A, McManus EL (2012). The use of spinal manipulative therapy for pediatric health conditions: a systematic review of the literature. *The Journal of the Canadian Chiropractic Association*.

[24] Choi EM, Jiang F, Longhurst JC (2012). Point specificity in acupuncture. *Chinese Medicine*.

[25] Deadman P, Al-Khafaji M, Baker K (2006). *A Manual of Acupuncture*.

[26] Yin J, Chen J (2010). Gastrointestinal motility disorders and acupuncture. *Autonomic Neuroscience*.

[27] Lian Y, Chen C, Hammes M, Kolster B (2008). *Seirins stora akupunkturatlas: en beskrivning av akupunkturpunkterna*.

[28] Jiming C, Xinming S, Junqi C (1990). *Essentials of Traditional Chinese Pediatrics*.

[29] Adams D, Cheng F, Jou H, Aung S, Yasui Y, Vohra S (2011). The safety of pediatric acupuncture: a systematic review. *Pediatrics*.

[30] Raith W, Urlesberger B, Schmölzer GM (2013). Efficacy and safety of acupuncture in preterm and term infants. *Evidence-Based Complementary and Alternative Medicine*.

[31] Li H, Wang YP (2012). Effect of auricular acupuncture on gastrointestinal motility and its relationship with vagal activity. *Acupuncture in Medicine*.

[32] Jindal V, Ge A, Mansky PJ (2008). Safety and efficacy of acupuncture in children: a review of the evidence. *Journal of Pediatric Hematology/Oncology*.

[33] MacPherson H, Altman DG, Hammerschlag R (2010). Revised STandards for Reporting Interventions in Clinical Trials of Acupuncture (STRICTA): extending the CONSORT statement. *Journal of Evidence-Based Medicine*.

[34] Scheid V, Ward T, Tuffrey V (2010). Comparing TCM textbook descriptions of menopausal syndrome with the lived experience of London women at midlife and the implications for Chinese medicine research. *Maturitas*.

[35] Birkeflet O, Laake P, Vøllestad N (2012). Traditional Chinese medicine patterns and recommended acupuncture points in infertile and fertile women. *Acupuncture in Medicine*.

[36] Birkeflet O, Laake P, Vøllestad N (2011). Low inter-rater reliability in traditional Chinese medicine for female infertility. *Acupuncture in Medicine*.

[37] SBU

